# Male Breast Cancer in Togo: Imaging and Clinicopathological Findings

**DOI:** 10.1155/2020/3056067

**Published:** 2020-08-31

**Authors:** Tchin Darre, Mazamaesso Tchaou, Toukilnan Djiwa, Panakinao Simgban, Ayi Kossi Amavi, Bidamin N'Timon, Abdoulatif Amadou, Mayi Bombonne, Bagassam Sama, Koffi Amégbor, Gado Napo-Koura

**Affiliations:** ^1^Department of Pathology, University Teaching Hospital of Lomé, Togo; ^2^Department of Imaging, University Teaching Hospital of Lomé and Kara, Togo; ^3^Department of Surgery, University Teaching Hospital of Lomé, Togo

## Abstract

**Background:**

Breast cancer in men is a rare condition, often diagnosed late. The purpose of this study was to describe its epidemiological, histopathological, and radiographic aspects in Togo.

**Materials and Methods:**

This was a descriptive retrospective study on cases of breast cancer in humans diagnosed histologically at the Laboratory of Anatomy Pathological and Imagery of the University Hospital in Lomé, over a period of 25 years (1995 to 2019). The parameters studied were epidemiological, anatomopathological, and imaging.

**Results:**

Eighty-two (82) cases were diagnosed, an annual frequency of 3.28 cases. The mean age was 45 ± 2.5 years; the range was 27-63 years. The family history of 47 patients (57.32%) was known. Carcinomas represented the predominant histological group with predominantly nonspecific invasive carcinoma (87.5%). These cancers were diagnosed at late stages (75.71% grade II). They were mainly of luminal B profile (38.75%) and associated with mutations of the BRCA2 and BRCA1 genes in 14.63% of the cases. The lesions were classified ACR 5 in 61.5% (11/18). Two cases of breast angiosarcoma were diagnosed by the identification of CD31 markers and factor VIII in immunohistochemistry. Hormone therapy such as tamoxifen was prescribed in all luminal patients (43 patients). Radiotherapy was administered to 15 patients (18.3%), with acute toxicity in 20% of the cases. After a median follow-up of 36 months, the evolution was complete remission in 27 patients (32.93%).

**Conclusion:**

Breast cancer in men is rare, often diagnosed late with a poor prognosis.

## 1. Background

Human breast cancer is a rare malignant tumor that accounts for less than 0.2% of all human cancers and less than 1% of breast cancers [1, 2]. However, its incidence has increased over the past 25 years as the world's population ages [3, 4]. Although its etiology is still little known because of its rarity, current knowledge incriminates the constitutional, environmental, hormonal (estrogen/androgen balance), and genetic risk factors (family history, Klinefelter syndrome, gene mutations BRCA1, and especially BRCA2) [1, 4, 5]. It generally occurs in elderly subjects and is associated with a poor prognosis because of an often late diagnosis [6, 7]. The mainstay of treatment consists of a modified radical mastectomy, with axillary dissection to assess the stage, prognosis, and the need for adjuvant treatment [8]. When matched for age, tumor size, grade, and axillary lymph node status, the prognosis is similar for men and women [8]. The objective of this study is to describe the epidemiological, anatomopathological, and imaging aspects of breast cancer in humans in Togo.

## 2. Materials and Methods

We report the results of a retrospective and descriptive study on cases of breast cancer in humans diagnosed histologically at the Pathological Anatomy and Imaging Laboratory of the Sylvanus Olympio University Hospital in Lomé, from January 1, 1995 to December 31, 2019, a period of 25 years. Togo is a small country of 56,600 km^2^, with an estimated population of 7,200,000, located between Ghana in the west and Benin in the east. These cases were collected from the registers of the said laboratory. The study material consisted of biopsies and operating pieces fixed in 10% buffered formalin and came from various health structures in the country. The parameters studied were epidemiological (frequency, age, location, family history, and circumstances of discovery) and pathological (nature of the sample, histological group, histological type, histoprognostic grade, pTNM stage (AJCC, 2017), molecular profile, and existence of mutations in the BRCA2 and BRCA1 genes). The imaging aspects observed during ultrasound and mammography in patients with an imaging record were taken into account. The size, American College of Radiology (ACR) classification, and axillary lymphadenopathy were studied.

Clinical, therapeutic, and prognostic data were collected from the patient's medical records.

The histologic analysis has been performed on paraffin-embedded and formalin-fixed specimens stained by hematoxylin-eosin-saffron. The immunohistochemical analysis has also been performed on paraffin-embedded and formalin-fixed tissues.

### 2.1. Immunohistochemistry Tests

Immunostaining was done for estrogen receptor (ER), progesterone receptor (PR), human epidermal growth factor receptor 2 (HER2), and Ki-67 count using a Ventana Benchmark immunostainer using the manufacturer's supplied antibodies. Positive status for ER and PR was defined as having nuclear staining in at least 10% of invasive tumors cells. HER2 was scored based on a 0 to 3 scale according to the criteria set by ASCO (American Society of Clinical Oncology). In the final analysis, a score of 3+ was considered overexpressed or positive and a score ≤2 as negative. Fluorescence in situ hybridization for HER2 amplification was not performed.

Breast cancer subtypes were defined according to the IHC expression of ER/PR/HER2 and Ki-67 count as follows: luminal A (ER+/PR+, HER2-, Ki‐67 ≤ 14%), luminal B (ER+/PR+, HER2+ or ER+/PR+, HER2-, Ki‐67 > 14%), HER2 enriched (ER-, PR-, HER2+), and triple negative (ER-, PR-, HER2-).

## 3. Results

### 3.1. Sociodemographic Data

Table 1 shows the sociodemographic characteristics. We identified a total of 82 cases of human breast cancer representing an annual frequency of 3.28 cases. The average age of the subjects was 45 ± 2.5 years; the range was 27-63 years. Thirty-eight (38) patients were under 40 years of age representing 46.34% of all cases, 32 patients (39.03%) were between 40 and 60 years of age, and 12 patients (14.63%) were over 60 years old. There were 41 cases (50%) in the left breast, 40 cases (48.78%) in the right breast and 1 case (1.22%) with bilateral location. Family history was known in 47 patients (57.32%). There were 27 cases with family history of breast cancer, 15 cases with family history of ovarian cancer, and 5 cases with family history of associated breast and ovarian cancer.

### 3.2. Clinical Data

The circumstances of discovery consisted of palpable mass in 68 cases (82.93%). It was an isolated palpable mass in 50 cases, associated with changes in the skin covering in 14 cases and lymphadenopathy in 4 cases. These skin changes were represented by an “orange peel” thickening in 11 cases and by a skin ulceration in 3 cases. The rest of the circumstances of discovery consisted of an isolated modification of the skin covering in 6 cases (5 cases of thickening with an “orange peel” appearance and 1 case of skin ulceration), a fortuitous discovery in 4 cases, and discharge bloody nipple in 2 cases.

### 3.3. Imaging Data

Eighteen medical imaging records were found, with at least one ultrasound performed on all 18 patients. A mammogram was coupled with ultrasound in 10 patients (55.6%). No other imaging modality was found. Eleven (61.1%) had had an ultrasound-guided microbiopsy. The lesions were classified ACR 5 in 61.5% (11/18), ACR 4 in 33.3% (6/18), and ACR 3 in 5.6% (1/18). The lesions had an average size of 29.2 mm ± 15.6 mm and extremes of 15 mm to 65 mm. Axillary lymphadenopathy was found in 44.4% of the cases.

### 3.4. Histopathological Data

The diagnosis of breast cancer was made on 14 biopsy fragments and 68 surgical pieces including 64 pieces of nodulectomy and 4 pieces of mastectomy with axillary dissection. The nodulectomy pieces were between 2 and 5 cm in size, ill-defined, firm in consistency, and showing foci of hemorrhagic changes. The mastectomy pieces all presented foci of necrotic and hemorrhagic changes with a tumor size varying from 3 to 6 cm. The examination of the axillary dissections found more than 3 lymph nodes in 75% of the cases. We found 80 cases (97.56%) of breast carcinomas and 2 cases (2.44%) of breast angiosarcoma were diagnosed by CD31 markers and factor VIII immunohistochemistry. Invasive carcinoma of the nonspecific type represented 87.5% (70 cases) of all carcinomas, followed by carcinoma in situ of the ductal type with 8 cases (10%) and 2 cases (2.5%) of papillary carcinomas. The diagnosis of invasive carcinoma of a nonspecific type was made on 60 surgical pieces including the 4 pieces of mastectomy and 10 biopsy fragments. By the Nottingham histoprognostic classification, 53 cases (75.71%) of these invasive carcinomas corresponded to grade II and 7 cases (10%) to grade III. Fifty-six (56) cases or 80% of these invasive carcinomas corresponded to the T2NxMx grade, 4.28% (3 cases) to the T2N2Mx grade, and 1 case (1.43%) to the T2N1Mx grade. The diagnosis of the 2 cases of mammary sarcoma was made on parts of nodulectomy. They both corresponded to grade 2 of the FNCLCC classification. Regarding molecular classification, 66 cases (82.5%) of these carcinomas expressed hormone receptors. Thirty-one cases (38.75%) had a luminal B profile; 13 cases (16.25%) had an enriched HER2 profile. The luminal A profile was seen 12 cases (15%), and 10 cases (12.5%) were triple negative. The search for mutations in the BRCA2 and BRCA1 genes was carried out in 12 patients. There were mutations in the BRCA2 gene in 9 cases (10.97%) and in the BRCA1 gene in 3 cases (3.66%). The histopathological characteristics are summarized in Table 2.

### 3.5. Therapeutic and Prognostic Data

Four (4.87%) patients underwent a mastectomy with lymph node dissection. Radiotherapy was delivered in 15 patients (18.3%), with a dose of 50 Gy on the wall. Acute toxicity from radiation therapy like radiodermatitis was found in 20% of cases. Sequential chemotherapy was administered as a neoadjuvant in 3 patients (3.66%), as an adjuvant in 12 patients (14.63%). Nausea-vomiting was the main side effect of chemotherapy (85% of cases). Hormone therapy such as tamoxifen was prescribed in all luminal patients (43 patients). Togo did not have treatment with trastuzumab before 2015; only 5 patients out of 13 in the enriched HER2 category were able to benefit from this treatment. After a median follow-up of 36 months, evolution was characterized by complete remission in 27 patients (32.93%), local relapse in 18 patients (21.95%), metastatic relapse in 9 patients (10.97%), and death in 7 patients (8.54%); 21 patients were lost to follow-up (25.61%). The bone was the main metastatic site with 55.56% of cases (5/9), followed by the lung with 22.22% of cases (2/9) and liver and skin with 11.11% each (1/9). The overall 5-year and 10-year survivals were 59% and 47%, respectively.

## 4. Discussion

We found an annual frequency of 3.28 cases of breast cancer in men, significantly lower than that of breast cancer in women in our country [9]. Breast cancer in men accounts for less than 1% of breast cancer diagnoses in the United States [10]. Breast cancer in men is a rare malignant tumor that represents less than 0.2% of all cancers in humans and less than 1% of breast cancers [1, 2]. Our study shows that breast cancer occurs in young adult men. This finding was also made in a study done on female breast cancer. Indeed, the African population in general and that of Togo in particular is young and would explain in part its average ages [8, 9]. The young age of our subjects could perhaps also be explained by the existence of genetic mutations in this population. Breast cancer in men due to a mutation in the BRCA2 gene occurs earlier and with a poorer prognosis [11]. It is estimated that around 10% of men with breast cancer have a genetic predisposition, with mutations in the BRCA2 gene being more clearly associated [12]. Mutations in the BRCA1 gene are also weakly associated [13]. In men with BRCA2 mutations, the estimated risk of developing breast cancer is 5% to 10% compared to a risk in the general population of 0.1% [10]. The estimated risk of developing male breast cancer associated with mutations in the BRCA1 gene is 1% to 5% [11, 13]. Thus, genetic counseling should be offered in each case. It is not rare to have bilateral breast cancer, although not as common as unilateral in men [1]. Indeed, we only found one case of bilateral localization. While gynecomastia is often bilateral and centered in the subareolar region, breast cancer in men is often unilateral and located eccentric to the nipple [14]. A family history of breast and/or ovarian cancer was present in 57.32% of our patients. About 15% to 20% of men with breast cancer report a family history of breast and/or ovarian cancer [14]. Family history increases the risk of breast cancer as it does in women. According to Weiss et al. [15], a family history of breast cancer in a first-degree man or woman multiplies the risk by two to three.

The circumstances of discovery were mainly represented by a palpable breast mass with 82.93% of all cases. It was isolated or associated with changes in the skin covering, with lymphadenopathy. In a review of the literature on male breast cancer case reports, Senger et al. note that the majority of patients had a palpable mass clinically [16]. Other presentations included thickening of the skin, nipple retraction, and lymphadenopathy [2]. Breast cancer in men presents in most cases as a subareolar swelling, nipple retraction, or bloody discharge [8, 9].

Several imaging methods can be used in the male subject for breast exploration, but ultrasound is the basic examination. Due to the low abundance of breast tissue, mammography can be difficult to perform in the male subject [17]. Some studies have even found that ultrasound and clinical examination may be enough to decide what to do with a breast lesion in the male subject [18]. In Africa, there are a few studies on the radiopathological correlation of lesions suspected of breast cancer in men, such as that of N'timon et al. [19] in Togo and Ouedraogo et al. [20] in Burkina Faso. N'timon et al. in their study only considered the correlation of ACR4 and 5 lesions in imaging and the histological results of ultrasound-guided microbiopsies. The present study takes into account all aspects of imaging of lesions described as malignant in histology, whatever the nature of the part (microbiopsy, surgical biopsy, or surgical excision). This explains the presence in the series of cases of ACR3 lesion. Breast nodules classified ACR3 have a positive predictive value of less than 3% of malignancy [21]. This must also be taken into account in men, where the risk of error seems higher, which can lead to misunderstanding a malignant lesion for a simple gynecomastia due to the high prevalence of these types of lesion in men. In all cases, a unilateral gynecomastia in an elderly subject, in the absence of any hormonal or metabolic factor, must be verified by an ultrasound and a mammography and in case of doubt submitted for fine needle aspiration for cytology or surgical biopsy for histological diagnosis [18].

We observed a predominance of nonspecific invasive carcinoma with 87.5% of all carcinomas. Invasive nonspecific carcinoma is the main histological type of breast cancer in men [3, 4]. No lobular carcinoma had been found. Invasive lobular carcinoma is an exceptional diagnosis in the male breast [16]. The majority of these invasive carcinomas corresponded to grade II of the Nottingham classification (75.71%) and to grade pT2NxMx (80%) of the pTNM classification (AJCC, 2017). Breast cancer in men is often diagnosed at a late stage because it is mistaken for gynecomastia without histological confirmation [7, 22]. Compared to breast cancer in women, breast cancer in men more often expresses hormone receptors [22]. In our series, 82.5% of the cases expressed hormone receptors. A recent study has shown that 5% of breast cancers in men have an overexpression of HER2 [23]. In our series, 16.25% of the cases express the HER2 gene. Breast cancer in men seems to have a poorer prognosis than in women [8, 12]. Tumor sizes as well as lymph node involvement are two important prognostic factors in human breast cancer [9]. Men with a tumor of 2 to 5 cm have a 40% increased risk of death compared to those whose tumor is less than 2 cm in maximum diameter [10, 13]. Indeed, pure mesenchymal tumors of the breast, including angiosarcoma, are very rare [24, 25] in two distinct forms: a primary form that appears in the breast parenchyma occurring in young women between 20 and 40 years of age, without a history of prior neoplasm, and a secondary form that develops in the skin, chest wall, or breast parenchyma after surgery and postoperative radiotherapy for breast cancer [25]. It corresponds to 0.04% of p tumors and 8% of breast sarcomas [25].

In general, the prognosis for men with breast cancer is similar to that for women [9, 25, 26]. The therapeutic strategy for the management of cancers in men is similar to that in women [27, 28]. At the early stage, most men are treated with a modified radical mastectomy associated with axillary dissection or selective lymphadenectomy [21, 29, 30]. In a series of 31 cases of carcinoma in situ of the ductal type, Zhu et al. found 3 relapses after 6 lumpectomies against a single case of relapse for 25 mastectomies [22]. The small size of the male mammary gland makes it difficult to pass into healthy margins [30]. Lumpectomy is therefore not recommended [15, 21]. Conservative surgical treatment has no indication in the treatment of breast cancer in men because of the small breast volume and the easy acceptance of mastectomy by men [31]. Postoperative radiotherapy improves local control and progression-free survival but has no impact on overall survival [29, 32]. Fifteen (18.3%) of our patients received radiation therapy. Hormone therapy of the tamoxifen type is considered to be the therapeutic standard in the adjuvant setting in patients expressing hormone receptors [15, 32]. The main side effects are the risk of thromboembolic complications, hot flashes, and decreased libido [30]. Forty-three (43) of our patients received tamoxifen hormone therapy. Chemotherapy finds its place in patients who do not express hormone receptors or in case of resistance to first-line hormone therapy [29, 30]. Togo did not have treatment with trastuzumab before 2015; only 5 patients out of 13 in the enriched HER2 category were able to benefit from this treatment. The overall survival at 5 and 10 years of breast cancer in men is, respectively, around 60% and 40% according to Meriem et al. [31]. In our series, the 5-year and 10-year overall survivals were 59% and 47%, respectively.

## 5. Conclusions

Breast cancer in men is a rare condition, often diagnosed late and with a poor prognosis. Male breast cancer linked to a mutation in the BRCA2 gene occurs earlier and has a poorer prognosis. When matched for age, tumor size, grade, and axillary lymph node status, the prognosis is similar for men and women concerted efforts should be made to educate the public and healthcare professionals, in order to make diagnoses earlier and thus improve the prognosis.

## Figures and Tables

**Table 1:** Sociodemographic characteristics of patients.

	Value
Annual frequency	3.28
Age (year)	
(i) Average age	45 ± 2.5
(ii) Range	27-63
(iii) <40	38/82
(iv) 40-60	32/82
(v) >60	12/82
Location	
(i) Left breast	41/82
(ii) Right breast	40/82
(iii) Bilateral	01/80
Family histories	47/82
(i) Breast cancer	27/82
(ii) Ovarian cancer	15/82
(iii) Breast and ovarian cancer	05/82

**Table 2:** Histopathological characteristics of patients.

	Value
Nature of the sample	
(i) Biopsy	14/82
(ii) Operating sample	68/82
Histological groups and types	
(i) Carcinoma	80/82
(i1) Invasive ductal carcinoma, NOS	70/82
(i2) Ductal intraepithelial neoplasia	08/82
(i3) Invasive papillary carcinoma	02/82
(ii) Angiosarcoma	02/82
Elston and Ellis classification (Nottingham)	60/82
Grade III	07/82
Grade II	53/82
pTNM classification	60/82
(i) pT2NxMx	56/82
(ii) pT2N2Mx	03/82
(iii) pT2N1Mx	01/82
Molecular classification	66/82
(i) Luminal A	12/82
(ii) Luminal B	31/82
(iii) HER2 enriched	13/82
(iv) Triple negative	10/82
BRCA mutations	12/82
BRCA2	09/82
BRCA1	03/82

## Data Availability

All data generated or analyzed during this study are included in this published article.
